# Geneva Medical College

**Published:** 1848-12

**Authors:** 


*Geneva Medical College.*—We stated in a previous number, that the class in attendance at this Institution numbered *fifty-three.* This was the number at the commencement of the term. The number has since (as we are informed) increased to *sixty-three.*

